# Poster Session I - A71 ENDOSCOPIC ULTRASOUND-GUIDED THROMBIN INJECTION, A NOVEL THERAPY, IN A CHALLENGING CASE OF PANCREATIC PSEUDOANEURYSM

**DOI:** 10.1093/jcag/gwaf042.071

**Published:** 2026-02-13

**Authors:** N Ashrafinia, R Samji, C Wong, K Bishay

**Affiliations:** University of Alberta Faculty of Medicine & Dentistry, Edmonton, AB, Canada; University of Alberta Faculty of Medicine & Dentistry, Edmonton, AB, Canada; University of Alberta Faculty of Medicine & Dentistry, Edmonton, AB, Canada; University of Alberta Faculty of Medicine & Dentistry, Edmonton, AB, Canada

## Abstract

**Background:**

Visceral pseudoaneurysms are a rare but potentially fatal complication of chronic pancreatitis, with up to 90% mortality rate if left untreated. Currently, percutaneous and endovascular angioembolization are used in elective and emergency settings employing coils, glue, stents, and thrombin injections. Previously, surgery was the only option when percutaneous attempts were unsuccessful. Endoscopic ultrasound-guided (EUS) allows direct examination of visceral pseudoaneurysms with potential for endoscopic therapy. Limited literature has reported EUS-guided thrombin injection, particularly in challenging cases with poor visualization or endovascular inaccessibility.

**Aims:**

To describe a successful obliteration of a peri-pancreatic pseudoaneurysm using EUS-guided thrombin injection.

**Methods:**

A detailed chart and literature review were performed.

**Results:**

A 49-year-old male with a history of alcohol related pancreatitis presented with a 2-week history of melena and worsening of abdominal pain. On investigations, he had a hemoglobin drop (110 g/L-4 months prior, to 67 g/L) and elevated lipase (836 U/L). CT showed peripancreatic stranding with calcifications suggestive of acute-on-chronic pancreatitis, a small pseudocyst (3.0x2.9x4.2 cm) in the pancreatic tail, and a small adjacent well-circumscribed hyperattenuating focus (1.3x1.7x1.7 cm). The splenic vein appeared patent distally, with extensive SMV abdominal collaterals. Gastroscopy showed an oozing esophageal varix at the gastro-esophageal junction, requiring bandings to achieve hemostasis, and friable gastric mucosa with a fullness in the cardia that was felt to be gastroesophageal varix-type 1. CT angiography showed a small peripancreatic pseudoaneurysm from the small pancreatic arterial branch of the splenic artery (2.8x2.6x2.3 cm), significantly increased from the previous. Initial attempts at IR-guided intervention were unsuccessful due to a lack of access to the pseudoaneurysm. Given the proximity to the gastric body, EUS-guided thrombin injection was pursued; there was a 33x32 mm pseudoaneurysm adjacent to the pancreatic tail, showing a Yin-Yang sign on Doppler. With a 22-gauge Olympus EZ shot needle, the pseudoaneurysm was punctured. 350 IU of thrombin was injected into the pseudoaneurysm with excellent opacification and loss of Doppler flow. A small amount of thrombin was injected as the needle was withdrawn to minimize the risk of bleeding or extravasation into the tract. The patient remained stable with no complications. Subsequent imaging showed successful obliteration of the pseudoaneurysm.

**Conclusions:**

EUS-guided thrombin injection is a novel modality in pancreatic pseudoaneurysm management, particularly in cases with limited visualization or challenging vascular access. Larger studies with long-term follow-ups are required to further evaluate this technique.

**Funding Agencies:**

None

